# An Overview on the Alignment of Radiation Protection in Computed Tomography with *Maqasid al-Shari’ah* in the Context of *al-Dharuriyat*

**DOI:** 10.21315/mjms2023.30.3.5

**Published:** 2023-06-27

**Authors:** Siti Aisyah Munirah Bohang, Norhanna Sohaimi

**Affiliations:** Department of Diagnostic Imaging and Radiotherapy, Kulliyyah of Allied Health Sciences, International Islamic University Malaysia, Pahang, Malaysia

**Keywords:** radiation protection, computed tomography, Maqasid al-Shari’ah

## Abstract

The increasing utilisation of computed tomography (CT) in the medical field has raised a greater concern regarding the radiation-induced health effects as CT imposes high radiation risks on the exposed individual. Adherence to radiation protection measures in CT as endorsed by regulatory bodies; justification, optimisation and dose limit, is essential to minimise radiation risks. Islam values every human being and *Maqasid al-Shari’ah* helps to protect human beings through its sacred principles which aim to fulfil human beings’ benefits (*maslahah*) and prevent mischief (*mafsadah*). Alignment of the concept of radiation protection in CT within the framework of *al-Dharuriyat*; protection of faith or religion (*din*), protection of life (*nafs*), protection of lineage (*nasl*), protection of intellect (*‘aql*) and protection of property (*mal*) is essential. This strengthens the concept and practices of radiation protection in CT among radiology personnel, particularly Muslim radiographers. The alignment provides supplementary knowledge towards the integration of knowledge fields between Islamic worldview and radiation protection in medical imaging, particularly in CT. This paper is hoped to set a benchmark for future studies on the integration of knowledge between the Islamic worldview and radiation protection in medical imaging in terms of other classifications of *Maqasid al-Shari’ah*; *al-Hajiyat* and *al-Tahsiniyat*.

## Introduction

Ionising radiation in computed tomography (CT) imposes high radiation risks to patients. The increased utilisation of CT in the medical field worldwide raises greater concern regarding the radiation-induced health effects on the exposed individual. Adherence to radiation protection measures in CT as endorsed by regulatory bodies is essential to minimise radiation risks. Islam values every human being and *Maqasid al-Shari’ah* helps to protect human beings through its sacred principles. The alignment of the radiation protection concept in CT within the framework of *al-Dharuriyat*; protection of faith or religion (*din*), protection of life (*nafs*), protection of lineage (*nasl*), protection of intellect (*‘aql*) and protection of property (*mal*) is essential to protect human being.

### Overview of Computed Tomography

CT was introduced in the 1970s by Godfrey Newbold Hounsfield which generated cross-sectional images of human anatomy through a computer as a narrow beam of X-rays rotated around a patient’s body. The invention and advancement of CT have overcome limitations in plain or conventional radiography. These include slip ring technology in CT for continuous rotation of X-ray tube around a patient’s body which eliminates the superimposition of the anatomy of interest. CT image reconstruction software allows the reconstruction of 3D images with improved image quality. Post-examination manipulations, such as multi-planar reformation (MPR), 3D volume rendering (VR), surface shaded display (SSD), window setting, window width and window level, allow visualisation of CT images at different image planes, tissue characteristics, shades of grey and image brightness without exposing extra dose to a patient. CT number (Hounsfield unit [HU]) is very useful in differentiating normal and pathological tissues and also in characterising different types of tissue; bone, muscle, fat and soft tissue. The utilisation of CT technology in various clinical applications is also proven to reduce discomfort during a medical imaging examination, minimises post-procedural risks, is cost-effective, requires short acquisition time and improves spatial resolution. As a result, it has become a medical imaging modality of interest in various clinical applications worldwide such as the CT angiography (CTA) (1), CT Colonography (2) and CT-guided procedures; diagnostic or therapeutic (3), dual-energy CT (DECT) (4), hybrid PET-CT (5, 6) and cone-beam computerised tomography (CBCT) (7, 8). Increased utilisation of CT is a greater concern because CT is associated with a high radiation dose.

### Why Increased Utilisation of CT is a Global Concern?

CT scans are recognised to be the main contributor to higher radiation dose in medical exposures (9–13), particularly in multi-phasic CT procedures (14). This has caused a global concern over radiation protection issues in CT since exposure to ionising radiation, such as in CT, could lead to radiation-induced health effects. However, to date, there has been no conclusive data that corroborate the link between low radiation doses in CT to radiation-induced health cancer as the data have been extrapolated from atomic bomb survivors who had received much higher radiation doses (15). However, adhering to linear non-threshold (LNT) models; the radiation-induced health effects occur by chance (probability dependent on the dose), it is important to carry out endorsed and authorised radiation protection measures in CT into practice.

Ionising radiation imposes a greater concern to humanity as it has higher energy to ionise atoms and molecules as well as break chemical bonds within the irradiated materials (16). These cause the deleterious radiation-induced health effects that arise from ionising radiation which can degrade one’s quality of life. There are two categories of radiation effects on the human body: i) deterministic and ii) stochastic. Deterministic effects manifest over a known threshold and result in cell death, erythema, epilation, tissue necrosis and cataracts. Deterministic effects can be reduced by proper optimisation during the procedures such as tight collimation and application gonadal shielding. Stochastic effects occur due to chromosomal damage which can result in birth defects and they are recognised as a potential cause of carcinogenesis due to cell mutation (17). Unlike deterministic effects, stochastic effects do not have a known dose threshold and the occurrence of effects increases with the increment of radiation dose.

### Radiation Protection in Computed Tomography

The International Commission Radiation Protection (ICRP) has developed the radiation protection guidelines since 1920 and they have been defined as actions taken by radiation personnel to safeguard patients, personnel and the general public from unnecessary exposure to ionising radiation (18). The ICRP and International Atomic Energy Association (IAEA) have endorsed three general principles of radiation protection: i) justification; ii) optimisation and iii) dose limit. The implementation of these three principles of radiation protection measures in CT examination is as follows.

### Justification of Computed Tomography Examinations

In the Publication 87 (19), one of the principles in radiation protection; justification, it highlights that the request for a CT examination should only be done by qualified medical practitioners after discussions with radiologists. This is necessary to ensure that only a clinically justified CT examination can be conducted for the result to serve its purposes. Through justification criterion, the protocols of CT examinations for a particular indication will be determined, whether there is a need for standard-dose CT or low-dose CT (20). Different clinical indications require different CT protocols. In justification, the consideration to undergo other medical imaging modalities in the first hand, which is fewer radiation doses than CT such as plain radiography, will also be weighted. An unjustified repetition of CT examination should not be conducted and to avoid this issue, referring clinicians and radiologists must be aware and knowledgeable of the records of previous investigations. Thus, justification is the first level of radiation protection in CT to avoid unnecessary exposure towards patients.

### Optimisation of Computed Tomography Examination

The optimised process of conducting CT examinations in which a substantial portion of the standard dose is eliminated as low as reasonably achievable (ALARA) without degrading the diagnostic performance of CT images (21). Adjustments of CT examination parameters according to patient size and application of shielding are among the optimisation methods performed in CT. Radiology personnel, i.e. radiographers, should be knowledgeable to alter the CT examination parameters to patient size and indication accordingly. The optimisation should be implemented for all patients undergoing CT examinations without discrimination but focuses more on fragile populations, i.e. pregnant or breastfeeding women and children (22).

The age of CT scanners affects the radiation dose in CT. Older CT scanners are associated with higher radiation doses due to lack of advanced CT technology; automatic exposure control, automatic tube potential selection and iterative reconstruction (23–25). Modern CT scanners are also equipped with beam-shaping (bowtie) filters to improve image uniformity and reduce the patient radiation dose (26). Thus, the radiology department needs to update or change the CT modality after a certain period as it is necessary to produce the optimum quality of diagnostic CT images and protect patients from unnecessary radiation doses.

Patient stature or body habitus significantly influences the radiation dose in CT examinations. Different body habitus requires adjustment in CT imaging parameters to produce adequate power and amount of X-ray photon to penetrate a patient’s body as well as generate optimum diagnostic CT images. Failure to apply an appropriate adjustment of CT imaging parameter in accordance to patient body habitus results in unnecessary or excessive radiation dose towards patients. A patient with a larger body habitus requires higher penetrating power and amount of X-ray photons to penetrate the irradiated region of interest to generate optimum diagnostic CT images compared to leaner patients (27). CT imaging parameters in a paediatric patient should also be different from adults and follow paediatric needs. Various studies (28) have shown that paediatric patients receive six times higher radiation doses due to failure to adjust CT imaging parameters to the need of paediatric patients; small body habitus, long life expectancy and presence of numerous proliferating cells in their bodies. Thus, understanding the effect of body habitus towards radiation dose is important for radiographers to adjust the CT imaging parameters accordingly to comply with the ALARA concept.

CT imaging parameters are a technical factor manipulated throughout the CT examination based on patient characteristics and body habitus to yield optimum diagnostic CT images at lower radiation doses. The CT imaging parameters focused by the author are X-ray tube kilovoltage (kVp), X-ray tube current (mA), X-ray tube rotation time (sec), automatic tube current modulation (ATCM), pitch, scan range, phase of scans and iterative reconstruction (IR).

X-ray tube kilovoltage (kVp) is related to the intensity of the X-ray photons emitted from the X-ray tube. Higher X-ray tube kVp results in higher intensity of X-ray photon and has strong penetration power to penetrate a patient’s body and be detected by detectors. The kVp is exponentially related to radiation dose (29, 21, 30). Studies show that lowering 20 kVp will reduce the radiation dose by about 35%–40% and in paediatric (16), it has been mentioned that the selection of as low as 80 kVp can be conducted without degrading the image quality. Thus, the manipulation of kVp is essential to reduce the radiation dose exposed to the patient by taking into consideration the clinical indication and patient size.

Tube current (mA) influences the number of X-ray photons generated from the X-ray tube. An increase in mA increases the number of X-ray photons generated resulting in a higher CT radiation dose (21). Low mA increases image noise (quantum mottle) and degrades the diagnostic value of CT images.

mA works together with X-ray tube rotation time (s) and this is known as mAs (31). Thus, the manipulation of mAs should be conducted based on patient size. The latest technology advancement in CT makes the selection of mAs more effective and reliable by the ALARA concept. This is the automatic tube current modulation (ATCM) as explained below.

ATCM reduces radiation dose by automatically modulating the CT tube current according to patient size obtained through the localiser images (32). ATCM makes the selection of CT tube current more effective and accurate of patient size for radiation dose reduction. Various studies have shown that ATCM is efficient in reducing radiation dose and maintaining a certain level of diagnostic CT image quality (33, 34) as well as increasing the lifespan of CT X-ray tubes by reducing the load. There are several parameters during the acquisition of CT localiser images that need careful attention to optimise the utilisation of ATCM to reduce radiation dose. The parameters include projection angle of the localiser, patient-centred within the gantry, protocol selection, scanning direction, scan protocol (organ characteristic) and use of bismuth shielding. Thus, careful manipulation of CT acquisition parameters is fundamental to optimise ATCM to ensure the radiation dose complies with the ALARA concept.

Pitch refers to the movement of the table during one rotation of the CT X-ray tube over a slice of image collimation. Pitch determines the degree of overlap between the adjacent CT images and it is inversely proportionate with patient dose (35). There are three different ranges of pitch used in CT examinations. Pitch equals 1 (*P* = 1) means that CT images are adjacent with one another without overlapping or gapping. Pitch less than 1 (*P* < 1) means that CT images are overlapping with the adjacent CT images. Overlapped CT images increase in radiation dose as radiation is exposed to the same anatomical region repeatedly. Pitch more than 1 (*P* > 1) means that CT images are gapping with the adjacent CT images. Gapped CT images reduce radiation dose as it allows non-exposition of the same anatomical region repeatedly. This is due to an increase in the table speed and acquisition time. High pitch CT is widely used in cardiac imaging and paediatric imaging (36). Pitch helps to reduce radiation dose to the patient, thus, radiographers must make use of this parameter accurately to fulfil its purpose by considering the clinical indication and patient size.

Scan range means the length of the patient’s body in z-direction being scanned throughout a particular CT examination. Scan length in CT examination also influences the CT radiation dose. The larger the scan range involved in a particular CT examination, the bigger the anatomical region being exposed to radiation. Thus, to avoid exposing unnecessary or excessive radiation doses to patients, scan length should be limited to the clinical area of interest as mentioned in various studies (37, 38).

CT examination can be conducted either in a single-phase or multiphase depending on the clinical indication. A single-phase CT can be conducted with or without intravenous injection of contrast media. Basically, in a multiphase CT, there are three other enhanced phases: i) arterial; ii) venous and iii) delayed, scanned along with unenhanced phases and this makes multiphase scan high in radiation dose (14). Thus, the selection of phases of CT scan is important in radiation reduction measures and it is advisable to conduct single-phase especially in paediatric imaging (22) to reduce radiation dose. However, the decision also depends on the clinical indication of a particular CT examination.

The reconstruction algorithm does not directly affect the radiation dose but it affects the CT image quality. Manipulating certain CT parameters to reduce radiation dose affects a certain level of CT image quality. To compensate for these drawbacks, iterative reconstruction (IR) algorithms have been introduced to replace conventional filtered back projection (FBP) which has been used for decades as a CT image reconstruction software. FBP produces better image quality in a short reconstruction time. However, FBP depends on the number of X-ray photons reaching the detectors. Low X-ray photon detected on the detectors due to low dose of CT protocol results in poor quality of images reconstructed by FBP. IR overcomes the limitation of FBP by enabling the low-dose CT conducted with high-diagnostic quality and providing 40%–80% lower radiation dose (39–41). IR is also less computationally demanding and requires a short reconstruction time. Thus, the development of IR algorithms leads to more effective CT protection measures while sustaining optimum diagnostic CT images.

Shielding reduces radiation dose to patient as it acts to remove the lower-energy photons which do not contribute to the image formation from reaching the exposed body. Lead is a commonly used material for radiation shielding as it has a high atomic number and density to shield from the radiation. Other than lead shielding, one of the widely used shielding in CT nowadays is the bismuth shielding. Bismuth shielding is proven to reduce radiation dose without degrading the diagnostic image quality (42–44). The bismuth shielding should be placed properly on a patient’s body to avoid the formation of beam hardening effect on a CT image and allow for optimum utilisation of radiation shielding. Thus, radiation shielding should be used properly to fulfil its purpose as well as adhere to the ALARA concept.

### Dose Limit of Computed Tomography Examination

Application and adherence to dose limit/dose reference level (DRL) are essential to ensure the radiation dose exposed to a particular individual is within the acceptable and permissible range. Dose limits have been established by authorised bodies to reduce the risk of radiation-induced health effects that arise from exposure to ionising radiation. There are several dosimetry metrics used to measure the radiation dose in CT: volume CT dose index (CTDI) (45), dose length product (DLP), effective dose (ED) (46) and size-specific dose estimate (SSDE) (47).

DRLs are an important tool that helps in dose monitoring and standardisation of CT protocols for the same examinations between different CT centres (48, 49). Standardisation of CT protocols of the same CT examination between different CT centres reduces the radiation dose variation exposed to patients. CTDI (CTDI_vol_ and CTDI_w_) and DLP are the recommended dose indices for CT DRLs by ICRP and the European Commission (50).

## Overview of *Maqasid al-Shari’ah*

The knowledge of the Islamic has been discussed previously by various Islamic scholars. However, the term *Maqasid al-Shari’ah* has been introduced by *Imam al-Syafi’e* in his book; *al-Risalah*. This term has been used till the present day. Various Islamic scholars after him have reconstructed, refined and detailed the approaches in their writings, such as *al-Juwayni, al-Ghazali*, *Izz al-Din al-Salam* and *al-Syatibi*.

The word *maqasid* (singular *maqṣad*) is derived from the Arabic root of *qaṣada* which means purposes, objectives and principles. Muslim jurist, Imam Al-Shatibi (d. 1388) describes it as ‘the attainment of good, welfare, advantage, benefits and prevention of evil, injury or harm.’ (51). *Maqasid al-Shari’ah* is the divine wisdom of Allah behind all His laws to benefit the human being either in a general or specific situation (52, 53).

*Maqasid al-Shari’ah* is important for human survival and spiritual well-being either as an individual or within a whole society. This is because *maqasid* aims to fulfil human beings’ benefit (*maslahah*) and prevent mischief (*mafsadah*) based on the sacred revealed knowledge; Al-Quran and As-Sunnah (54). *Maqasid al-Shari’ah* does not protect the interest of human being based on human desires but based on divine revelations. Understanding the *Maqasid al-Shari’ah* in a holistic view is crucial as its principles explain the relationship between Islamic law, human rights, development and civilisation (53).

*Al-Juwayni* (55, 56) has classified *Maqasid al-Shari’ah* into three levels; i) *al-Dharuriyat* (necessities); ii) *al-Hajiyat* (needs) and iii) *al-Tahsiniyat* (luxuries) based on the levels of importance. *Al-Dharuriyat* is an important element in human life and the absence of these elements results in a matter of life and death. *Al-Dharuriyat* contains five objectives: i) protection of faith or religion (*din*); ii) protection of life (*nafs*); iii) protection of lineage (*nasl*); iv) protection of intellect (*‘aql*) and v) protection of property (*mal*). For instance, protection of faith (*din*) can be seen in protecting Islam from the attack of its enemy. *Al-Hajiyat* is the requirement for a human to live without facing difficulties and it does not involve a matter of life and death. Among the examples of *al-Hajiyat* are marriage and communication tools. *Al-Tahsiniyat* can be seen as desirable things that give perfection and add additional value to human life such as luxurious houses and transportation. The relationship between the three levels is that the *al-Tahsiniyat* complements the *al-Hajiyat* and the *al-Hajiyat* supplements the *al-Dharuriyat* (57). Thus, the level of *Maqasid al-Shari’ah* is classified based on the priority of human interest that needs to be preserved, coverage of preservation, as well as the status of preservation’s intention. All the categories in *Maqasid al-Shari’ah; al-Dharuriyat, al-Hajiyat* and *al-Tahsiniyat* complement each other, facilitating the good outcomes of life and preventing evil and destructive conducts.

### Integration of Maqasid al-Shari’ah in Radiation Protection in Computed Tomography

In medical imaging, several studies have also been conducted to integrate Islamic values, particularly *Maqasid al-Shari’ah*, with utilising ionising radiation in performing medical examinations (58, 59). The integration illustrates the concept and practice of radiation protection methods in a similar stance to what Islam recommends. However, these studies only focus on radiation protection in general without specialising in any specific medical imaging modality. CT is associated with high radiation doses compared to other medical imaging modalities. There is a presence of upgraded methods of radiation protection only applicable in CT. Thus, there is a need for a study of aligning *Maqasid al-Shari’ah* in radiation protection measures in CT.

Through this paper, the author intends to align the concept of radiation protection in CT within the framework of *Maqasid al-Shari’ah* in the context of *al-Dharuriyat*. This alignment will help to visualise the concept of radiation protection in CT within the fundamental Islamic objectives; *Maqasid al-Shari’ah (al-Dharuriyat)* and strengthen the concept and the practices of radiation protection in CT among radiology personnel, particularly Muslim radiographers. This paper also aims to provide supplementary knowledge towards the integration between Islamic worldview and radiation protection in medical imaging, particularly in CT. This also sets a benchmark for future studies on knowledge integration between Islamic worldview and radiation protection in medical imaging in terms of other classifications of *Maqasid al-Shari’ah*, i.e. *al-Hajiyat* and *al-Tahsiniyat*.

In this paper, the authors align the concept of radiation protection measures in CT with the first level of classification in *Maqasid al-Shari’ah; al-Dharuriyat*. *Maqasid al-Shari’ah* should be acknowledged as a whole framework to reflect the holistic view of Islam. However, it is the author’s opinion to focus on one part of *Maqasid al-Shari’ah; al-Dharuriyat*, to discuss and elaborate the discourse. This is because *al-Dharuriyat* is considered an essential element in human life and affects the matter of life and death. Acknowledging the importance of *al-Dharuriyat*, the author believes it is agreeable to be aligned with the concept of radiation protection measures in CT. The five fundamental objectives in *al-Dharuriyat* are the protection of faith or religion (*din*), protection of life (*nafs*), protection of lineage (*nasl*), protection of intellect (*‘aql*) and protection of property (*mal*). Various radiation protection measures in CT are discussed and aligned with these five fundamental objectives accordingly.

### Protection of Religion (din)

The highest preservation in *Maqasid al-Shari’ah* is the protection of religion; Islam. Religion is the essence of every human being and it is the pillar of one’s life. Religion composes the belief in God Almighty and abides by all divine rules and guidelines to guide one’s life towards righteousness. Without religion, human beings are going astray and away from righteousness. The occurrence of radiation-induced health effects influences the religious and spiritual values of the exposed person, particularly as a *Muslim*. The effects could be seen in the elements of hindering a Muslim from performing his/her physical worshiping *(ibadah)* at the best state due to poor physical, emotional and health conditions.

*Ibadah* is one of the important components in Islam and it is the essence of human creation; to worship Allah. *Ibadah* is related to five pillars of Islam: i) the profession of faith (*shahadah)*; ii) performing prayer *(shalat)* five times daily; iii) payment of alms *(zakat)*; iv) fasting *(sawn)* in Ramadan and v) performing pilgrimage *(Hajj)* to those who can afford in terms of wealth and health. Radiation-induced health effects result in the poor physical condition and this hinders a Muslim to perform his/her *ibadah* at the best state. *Shalat* involves a certain level of physical activity such as standing, bowing prostration, and sitting consecutively and these require good physical health. The study also shows that Muslims who have cancer cannot fully fast in Ramadan (60) due to their poor health condition and the need to take prescribed medications. Even though Islam permits *rukshas*; leniency in performing *ibadah* when there are difficulties, but these somehow affect the satisfaction of Muslims in performing the *ibadah*.

In conclusion, protecting a person from radiation-induced health effects is important to protect the religion by allowing them to perform *ibadah* at their best health and satisfaction. Radiation protection measures in CT examinations are coherent and align with the essence of protection of religion *(din)*. Thus, Muslim radiographers should enforce and practice correct radiation protection measures in CT examinations to ensure that they are performing the responsibility in their profession as well as conducting *ibadah* towards Allah. The protection of religion can also be complemented by observing all four other protections.

### Protection of Life (Nafs)

Radiation dose from CT examination is a few times greater than conventional X-ray and can cause deleterious radiation-induced health effects. These health issues reduce ones’ quality of lives and for some if they are not treated at an early stage, the risk of mortality increases. Islam values one’s life as a sacred gift from Allah and *Maqasid al-Shari’ah* upholds the value of ‘life-saving’. The context of ‘life-saving’ is not only limited to the scope of protecting someone from mortality but also preserving the quality of one’s life at an optimum level. Exposure to high radiation doses in CT either to the patient or medical practitioners can harm one’s life.

CT examinations have adhered to the concept of benefit (detection of diseases) that outweighs harm (radiation risks). The benefit of diagnosing the diseases outweighs the health-related risks from ionising radiation as failure to detect the disease leads to the increase in severity of diseases and increases the risk of morbidity and mortality of a person (61). Detection of diseases, such as cancer, at an early and treatable stage, helps to reduce the burden faced by patients and improves their quality of life. Performing low radiation CT examination with poor CT image quality is also not accepted and is regarded as not protecting one’s life. This is because poor CT image quality does not have adequate diagnostic information to detect the disease as justified and increases the probability of one’s morbidity and mortality.

*Maqasid al-Shari’ah* acknowledges the detection of disease through CT examination as protection of life *(nafs)* to reduce the risk of morbidity and mortality due to early detection of diseases. Optimisation in conducting CT examinations and adherence to dose limits are complementary methods to *nafs* from radiation-induced health effects. Therefore, radiation protection measures in CT align with what Islam prioritises; *nafs*.

### Protection of Lineage (Nasl)

Lineage means a direct descendent from an ancestry. Protection of lineage prevents the successors of a person from any harmful and vicious events and also allows continuity of succession in a healthy and quality way. A productive succession is important in building a strong and productive civilisation as children are the asset of the future.

Utero-irradiation causes catastrophic radiation-induced health effects such as central nervous system abnormalities (neuropathology), cataracts, growth retardation, malformations, leukaemia, cancer and potential hereditary effects (62). Foetus’ exposure to ionising radiation leads to genetic damage due to chromosomal abnormalities within the foetus itself and transmits genetic damage to the future generation. Genetic damage is one of the stochastic effects of ionising radiation which has no dose thresholds and it is possible to happen even at low doses. The possibility for genetic damage increases linearly with the intensity of the radiation dose being exposed towards the foetus. The time of exposure relative to the conception also contributes to the severity of the genetic damage, particularly in the first trimester (63).

Aside from utero-irradiation, various studies show that infants and young children are at a greater risk for radiation-induced health problems (62, 64, 65) compared to adults (17, 28). This is due to the longer life expectancy of children which allows more time for the solid cancerous tissue to manifest itself. Besides, another factor for their sensitivity is a higher amount of proliferating and dividing cells among children as they undergo a growth state. Females are more sensitive towards the effects of ionising radiation compared to their counterparts (66) as females have more radiosensitive organs; genitalia and breasts.

Protection of lineage *(nasl)* within the context of CT radiation protection can be done through strict implementation of radiation protection measures for childbearing-aged women and children. Female patients within the childbearing age period are obligated to inform the radiographer about their last menstrual period (LMP) as LMP is used to determine whether a particular female patient is pregnant or not before undergoing any radiological examination using ionising radiation. Aside from LMP, protecting the radiosensitive organs, such as genitals and breasts, is also important to shield radiosensitive organs from radiation risk. For children, CT imaging techniques should be tailored according to patient size and age.

### Protection of Intellect (‘Aql)

Intellect is a special gift bestowed by Allah to all human beings which enables them to perform their roles as the vicegerent of Allah on earth. Intellect allows human beings to think, learn, reason, as well as remember to conduct daily activities (67). Protection of intellect is crucial as healthy intellectual humans tend to have a better quality of life and productive social functioning. Intellect is protected by conducting a healthy lifestyle, seeking knowledge, and participating in a cognitively stimulating activity (68).

Ionising radiation in CT is considered one of the highest radiation doses in medical exposures. Exposure to radiation in CT-induced radiation is related to psychological stress and anxiety which impacts the intellectual of the exposed person.

Psychological stress happens due to anxiety and excessive worry of the uncertainty of the dose received and health risks from ionising radiation either towards themselves (exposed persons) or their descendants (69, 70). It occurs regardless of any level of doses received and can cause short-term (71) as well as lifetime intellectual dysfunctions.

Protection of intellect *(‘aql)* can be implemented through strict adherence to radiation protection measures in CT to reduce the stress and anxiety which stem from radiation exposure in CT. These psychological effects can be reduced through effective communication and information transfer between radiographic personnel and patients. Thus, protecting the intellect *(‘aql)* through the above-mentioned methods can restore and preserve healthy intellectual functions and provide a better quality of life.

### Protection of Wealth (Mal)

Financial or wealth is one of the components that influences the quality of life in a particular person. Radiation-induced health effects especially cancers; leukaemia, thyroid and brain cancers cause financial difficulty to the patients and caregivers. Cancer is a long-term illness that is associated with a high financial cost including direct and indirect costs.

The direct cost is the expenditure that is directly involved with the treatment of cancer such as the costs of hospitalisation, medication, therapies and consultation with physicians. Cancer requires long-term treatment and therapies; radiotherapy and chemotherapy, and depends on expensive medications (72). Indirect cost refers to loss of resources due to unemployment as cancer affects the physical and psychological status of the patient and this influences the employment’s quality and performance. Poor health conditions cause higher absenteeism from work to extend loss of work, poor quality of work due to the side effects of therapies, reduction in income and saving (73). These direct and indirect costs are not only affecting the patient’s cancer but also their caregivers (74, 75). Unhealthy personnel and low productivity in employment lead to truncated profitability in the organisation and reduce the economic growth of a society and country.

Islam emphasises financial protection through the protection of wealth *(mal)* in *Maqasid al-Shari’ah* as it is essential to protect every Muslim from having financial burdens such as bankruptcy and huge medical debts that could reduce one’s quality of life, families, as well as progenies. Cancer is one of the examples of radiation-induced health effects arising from ionising radiation in CT that results in a high financial burden. Thus, protection of wealth *(mal)* can be aligned within the concept of radiation protection in CT in terms of implementation and application of the endorsed radiation protection measures in CT as previously mentioned.

## Conclusion

CT scans are recognised to be a high radiation dose modality among other modalities in medical imaging (76, 77). The increase in the number of CT examinations and increased dose per capita have brought global concern over radiation protection issues as exposure to ionising radiation such as in CT could lead to radiation-induced cancer. However, there have been no conclusive data that corroborate the link between low radiation doses in CT to radiation-induced cancer as the data have been extrapolated from atomic bomb survivors. However, adhering to linear no-threshold models, the radiation-induced health effects occur by chance (probability dependent on the dose), adherence to authorised radiation protection measures in CT is a better choice.

*Maqasid al-Shari’ah* is important for human survival and spiritual well-being as *Maqasid* aims to fulfil human beings’ benefit (*maslahah*) and prevent mischief (*mafsadah*) based on the sacred revealed knowledge; Al-Quran and As-Sunnah. Radiation protection measures in CT examination are endorsed for the protection of a person from the radiation-induced health effects which can cause harm and destruction to the person. These can be seen through the alignment of five fundamental principles of *Maqasid al-Shari’ah* in radiation protection measures in CT. First, protecting a person from the radiation-induced health effects is important to protect the religion, allowing them to perform *ibadah* at their best health and satisfaction. Second, acknowledging the detection of disease from CT examination and outweighing the risk of experiencing radiation-induced health effects acts as a protection of life *(nafs)*. Third, adherence to the endorsed and updated radiation protection measures in CT towards childbearing age women, pregnant women, foetus and children are one of the methods to execute protection of lineage *(nasl*). Fourth, protection of intellect *(‘aql)* is essential to reduce the probability of radiation-related psychological stress. Fifth, Islam emphasises financial protection through the protection of wealth *(mal)* to protect every Muslim from having financial burdens, such as bankruptcy and huge medical debt, that could reduce one’s quality of life, families, as well as progenies.

In conclusion, this paper has managed to align the radiation protection measures in CT examination according to Islamic essences and *Maqasid al-Shari’ah*. The alignment can strengthen the concept and practices of radiation protection in CT especially within Muslim professionals and provide supplementary knowledge to the existing studies on the integration of *Maqasid al-Shari’ah* radiation protection measures by specifically focusing on CT modality. It is hoped that this study will provide a benchmark for future studies to explore new knowledge of radiation protection that aligns with what Islam recommends.
